# Harnessing Pakistan’s youth for tobacco control

**DOI:** 10.12669/pjms.40.2(ICON).8953

**Published:** 2024-01

**Authors:** Saima Saeed, Kinza Zeeshan, Fizra Khan

**Affiliations:** 1 Dr. Saima Saeed, Indus Hospital & Health Network, Karachi, Pakistan; 2 Kinza Zeeshan, Indus Hospital & Health Network, Karachi, Pakistan; 3 Fizra Khan, Indus Hospital & Health Network, Karachi, Pakistan

As Pakistan gears up for the next Global Youth Tobacco Survey (GYTS), a brief review of the previous survey makes for grim reading; 10.7% of students have used any tobacco products, 87.5% obtained cigarettes from stores, shops, street vendors or kiosks, and more than 3 in 10 noticed advertisements when visiting points of sale.^1^ Yet, with a youth population of more than 64%,^2^ there are excellent opportunities to involve the nation’s young in tobacco control. Some examples of local youth-led initiatives include the LEAPS program, where trained 18- to 24-year-old females provide early childhood care and education to children under six years, improving the latter’s school readiness.^3^ Similarly, The Girl Child Project, with policy level stakeholder engagement and pre-implementation situational analysis, sensitized over 500 young females about gender issues through activities, motivational messaging and advocacy materials, thereby improving self-confidence and self-awareness.^4^ Galvanizing the youth by improving tobacco awareness is a popular, cost-effective^5^ intervention in public health spheres. For example, the Centre for Disease Control in the United States used social media, digital advertising, and educational resources for teachers and parents to prevent youth from using tobacco products through the Tobacco-free Teens campaign.^6^

Voices Against Tobacco (VAT) was launched in April 2021 by Indus Hospital & Health Network as a platform for young people to speak out about tobacco control issues. The premise is that youth empowerment will produce more sustainable outcomes in tobacco control, both for the youth concerned and the organization within which they function. VAT addresses different tobacco control measures by adopting youth-led collective advocacy methods. School sensitization and awareness sessions, empowering 1600 students on tobacco products, to date including novel tobacco and nicotine products, are performed. An integrated, participatory online ambassadorship program across Pakistan is employed; and last year VAT produced 23 active advocates through this. Other youth activities include in-person and online panel discussions with experts as well as poster competitions and short video reporting. Youth campaigning alone garnered 961 community petition signatures to raise taxes on tobacco last year.

An important intervention was conducting youth-led community surveys. Master trainers visited schools in Islamabad and Karachi arranging workshops with teachers on student leadership, community engagement and facts around tobacco control policies. Teachers went on to empower schoolchildren with these skills and knowledge, as a prelude to a pre-designed survey on tobacco. The exercise engaged 65 student ambassadors from Punjab Group of Colleges (2021) and 21 students from Sindh Madrassa tul Islam University (2023). These students further engaged 1,233 and 310 households respectively. The survey captured perceptions about impacts of tobacco, use of novel tobacco products, tobacco advertisement, its affordability and tobacco control policy measures.

The results showed almost half of respondents believed that tobacco products are extremely hazardous, generalizable to both cities. Meanwhile, 51% of respondents had tried some form of novel tobacco product. In Islamabad, 73% of respondents had seen some form of tobacco advertisement, whilst in Karachi, 55% had seen tobacco advertisement mainly on shops, markets, and point of sale displays. When asked about tobacco affordability and its influence on children, more than 70% respondents in both cities agreed that prices made tobacco accessible to the youth. Up to 67% in Karachi and 76% in Islamabad of respondents agreed that the government is not taking sufficient measures to decrease consumption of tobacco products in the country.

These tobacco youth surveys were not designed to achieve standard research criteria, e.g., predetermined sample size, controlling for socio-economic status and standardizing data collection. However, their main function in empowering the youth to speak in their communities about tobacco control policy was achieved. They also demonstrate the willingness of the public to support stronger tobacco control policies. VAT went a step further when in Islamabad it arranged a local seminar and invited the young ambassadors to share their results with policy makers directly, thus emphasizing advocacy impact.

Tobacco advocacy is an ongoing process and requires an integrated and collaborative approach with all relevant stakeholders and engaging the youth can play a pivotal role. In the months after the Islamabad seminar, the Government of Pakistan made important steps by increasing taxes on tobacco products in the 2022-23 Finance Bill, with further rises in subsequent budget reviews. There is more to be done in the landscape of tobacco control in Pakistan, such as controlling novel products, advertising and providing national cessation programs. Nonetheless, Voices against Tobacco, representing the will of Pakistan’s youth, has made an important contribution to a tobacco-free future in our country.

## Authors’ Contribution:

**SS:** Concept, manuscript editing.

**KZ and FK:** Data collection and manuscript drafting.
